# Within-species lateral genetic transfer and the evolution of transcriptional regulation in *Escherichia coli *and *Shigella*

**DOI:** 10.1186/1471-2164-12-532

**Published:** 2011-10-29

**Authors:** Elizabeth Skippington, Mark A Ragan

**Affiliations:** 1 The University of Queensland, Institute for Molecular Bioscience and Australian Research Council Centre of Excellence in Bioinformatics, Brisbane, Queensland 4072, Australia

## Abstract

**Background:**

Changes in transcriptional regulation underlie many of the phenotypic differences observed within and between species of bacteria. Lateral genetic transfer (LGT) can significantly impact the transcription factor (TF) genes which drive these transcriptional changes. Although much emphasis has been placed on LGT of intact genes, the units of transfer and recombination do not necessarily correspond to regions delineated by exact gene boundaries. Here we apply phylogenetic and network-based methods to investigate the relationship between units of lateral transfer and recombination within the *Escherichia coli - Shigella *clade and the topological properties of genes in the *E. coli *transcriptional regulatory network (TRN).

**Results:**

We carried out a systematic phylogenetic study of genetic transfer among 5282 sets of putatively orthologous genes from 27 strains belonging to the *E. coli - Shigella *clade. We then used these results to examine the evolutionary histories of TF genes, as well as the transcriptional regulation of lateral genes. We found evidence of LGT in 2655 (50.3%) gene sets: 678 (12.8%) show evidence of recombination breakpoints within the gene boundaries. Thus, within- and whole- gene lateral transfer is widespread among strains of *E. coli *and *Shigella*. We found that unlike global regulators, which have mostly evolved vertically, neighbour regulators (genes which regulate adjacent genes on the chromosome) have frequently been subject to transfer within the *E. coli - Shigella *clade. At least 56 (62%) of the 90 neighbour regulator gene sets examined show evidence of LGT, 19 (34%) of which have internal recombination breakpoints. Neighbour regulators show no evidence of co-transfer with their nearby target genes. Rather, the frequency of recombination breakpoints, and conflicting evolutionary histories among neighbour regulators and their target genes, suggest that the genomic regions encoding these genes have been constructed through successive layering of LGT events within the clade. We find no difference in the relative complexity of regulation (i.e. the number of regulators) of lateral versus vertical genes.

**Conclusions:**

Neighbour regulators show higher frequencies of transfer than other types of regulatory genes. This implicates the topological properties of regulatory genes in the TRN, and their physical proximity to targets on the chromosome, as contributing to successful LGT. The prevalence of recombination breakpoints within regulatory and target gene sets indicates that within-gene transfer has had a significant cumulative effect on the evolution of regulatory interactions in *E. coli *and *Shigella*.

## Background

Gene expression is regulated at multiple levels, from accessibility of DNA through the steps of transcription, post-transcriptional modification, translation and mRNA degradation. Transcriptional regulation is a particularly important stage for the control of phenotypic variation in response to internal or external signals. Transcriptional regulation is largely enacted by transcription factors (TFs) that bind to sites in genomes and, either alone or in combination with other TFs, thereby activate or repress the production of mRNA from one or more target genes. In *Escherichia coli *K12, 175 TFs which constitute more than 4% of the protein-coding gene complement have at least one annotated gene-regulatory interaction [1].

Many TFs fall within one of two broad classes, *global *or *local*, depending on the number of genes they regulate, the cellular processes in which they participate and their chromosomal location relative to their target genes [2,3]. Local regulators (also referred to as *neighbour *regulators) regulate a restricted number of genes that are in close physical proximity on the chromosome, whereas global regulators coordinately target large numbers of genes at multiple locations along the chromosome. Alternative criteria for defining global regulators have been presented [2,3].

Like other types of cellular interactions, the transcriptional regulatory interactions of an organism can be abstracted as a network in which molecules (genes or proteins) are represented as nodes (vertices) and regulatory interactions as edges. There are many ways in which transcriptional regulatory networks (TRNs) evolve, including *via *modifications to *cis*-regulatory regions of gene promoters [4], to the TF proteins themselves and to other *trans*-acting regulatory factors [5]. Here we focus on modifications to bacterial TRNs that have arisen *via *lateral genetic transfer (LGT).

Genomic studies leave no doubt that LGT has played a pervasive role in the evolution of prokaryotic genomes and is a significant source of phenotypic innovation among bacteria [6-9]. Successful LGT comprises a succession of steps: transfer and physical uptake of foreign DNA into a host new cell; recombination into the main chromosome, or maintenance on an extrachromosomal element; integration into genetic regulatory and biomolecular interaction networks; and finally, establishment in the host population [10]. Cellular networks, including TRNs, necessarily change and evolve as new genetic material appears and existing genetic material is overwritten or lost. In particular, newly introgressed lateral genes must recruit transcriptional regulators to become better integrated into host-regulatory networks and ensure appropriate stoichiometric and condition-dependent expression [11-13]. Recent analyses indicate that in *E. coli*, global regulators have mostly evolved vertically, whereas many local regulators have been acquired by LGT, often concurrently with the gene(s) they regulate [14]. In addition, *E. coli *genes of lateral origin exhibit more-complex regulation (tend to be regulated by more regulators) than genes which have been inherited vertically [14].

A major limitation of these and other previous studies of LGT and the evolution of transcription regulation is that they have taken whole genes as the unit of analysis, *i.e*. assumed that genes are transferred intact during LGT. In doing so, they have overlooked the potential significance of the transfer of within-gene fragments in the construction of genomic regions encoding *trans*-acting regulatory proteins and their targets. LGT does not necessarily involve genomic regions delineated by exact gene boundaries [15,16]. Chan *et al*. [16] reported clear evidence of one or more recombination breakpoints in at least 286 (19.6%) of 1462 sets of orthologous genes across prokaryotes, 134 of which did not show strong topological incongruence with the reference tree and, taking entire genes as the unit of analysis, would not be identified as lateral. A similar proportion of cryptic LGT was found among 13 *Staphylococcus *genomes [17]. Assigning entire genes as either vertical or lateral can thus significantly undervalue the contribution of LGT to the evolution of cellular networks [18].

Although LGT involving clusters of neighbouring genes and operons has been a focus of previous work [19], lateral transfer clearly need not involve genomic regions delineated by exact gene boundaries [15-17]. Are neighbour TF genes and their nearby target genes transferred and integrated all at once into the host regulatory networks, as has been suggested, or have these regions been built up via a succession of transfers?

Estimates of the frequency of LGT can be significantly affected by the number of genomes being compared and their relative genetic relatedness. Comparisons among strains of the same species can therefore be particularly informative [20]. The *E. coli - Shigella *clade has been well-sampled and annotated, with complete genome sequences of multiple diverse strains now available. Here we have analyzed 5282 sets of single-copy, putatively orthologous genes from 27 strains of *E. coli *and *Shigella *to determine the extent to which intact genes and within-gene fragments have been transferred and recombined *within *this clade. We report the frequencies of within- and whole-gene transfer in neighbour and global regulator gene sets, and examine evidence for propensity toward co-transfer of neighbour regulators and their nearby target genes. Finally we explore the regulation of lateral genes, comparing the regulation of genes which have been transferred and recombined intact with the regulation of genes within which gene fragments have been recombined in LGT.

## Results

### Lateral genetic transfer in *E. coli *and *Shigella*

We began our investigation of the impact of within-species LGT on the evolution of the *E. coli *TRN with a systematic study of genetic transfer more broadly among strains belonging to the *E. coli - Shigella *clade. We analyzed 27 completely sequenced genomes: 20 *E. coli *genomes and seven genomes belonging to the closely related genus *Shigella*. While *Shigella *species have historically been classified within a separate genus, it is now generally accepted that the *Shigella *phenotype evolved multiple times from different *E. coli *clones and therefore are part of the *E. coli *species [21-25]. *E. coli *strains have previously been divided into five distinct ECOR phylogenetic groups (A, B1, B2, D and E) based on genetic markers [26], and our dataset includes representative from each of these five groups.

**Figure 1:**
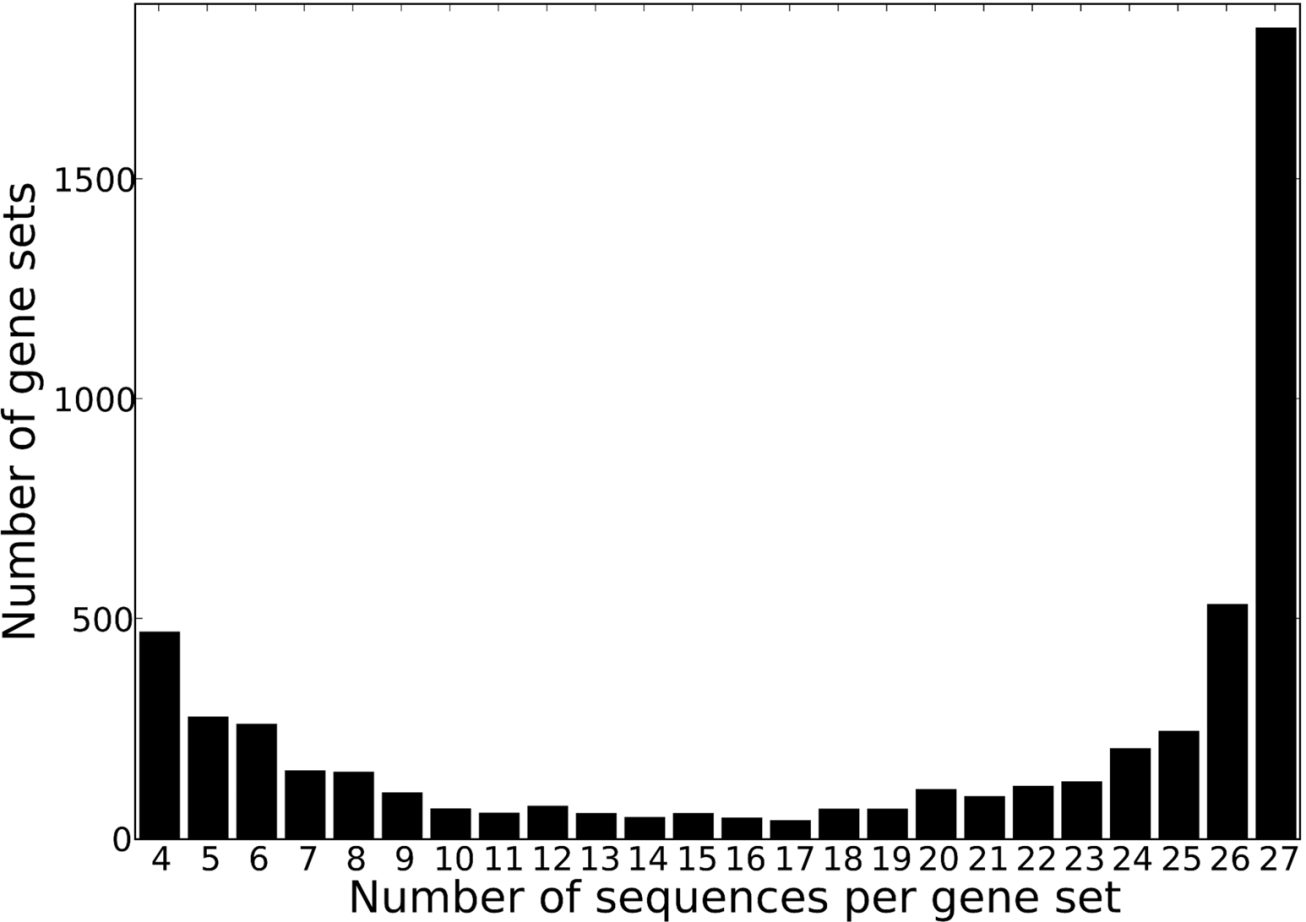
**Size distribution of 5282 *E. coli - Shigella *gene sets**.

**Figure 2:**
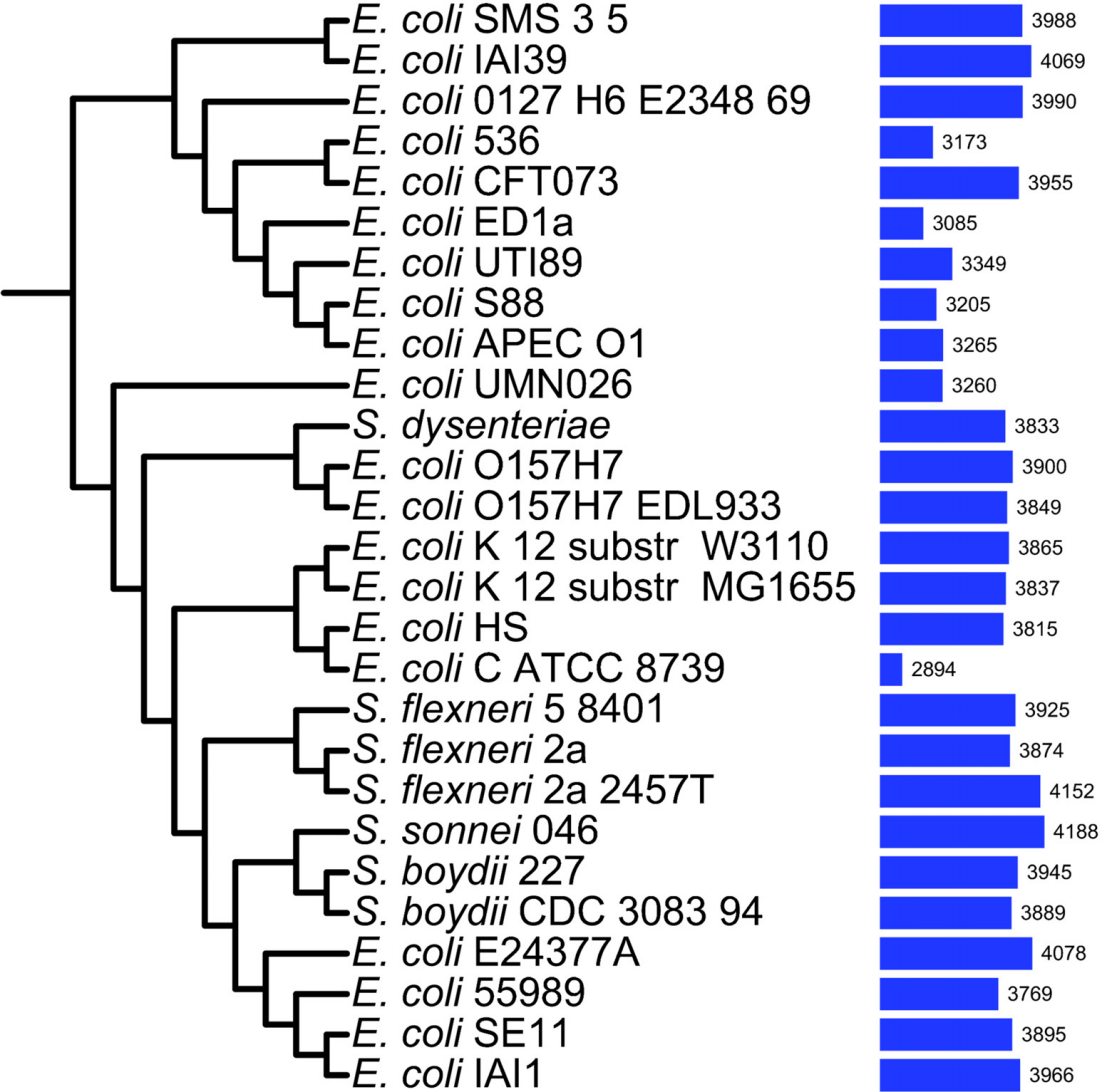
**Representation of 27 *E. coli *and *Shigella *strains within 5282 *E. coli *- *Shigella *gene sets**. *E. coli - Shigella *reference tree on the left was reconstructed from 5282 Bayesian protein trees using matrix representation with parsimony (MRP) [31]. Bars represent the number of gene sets which contain a gene from the corresponding genome.

We extracted 5282 positionally homologous gene sets of size *N *≥ 4 from a whole-genome alignment of the 27 *E. coli *and *Shigella *which was generated using the progressiveMauve program included in MAUVE version 2.3.0 [27]. Positional homology is implied among aligned regions of the genomes, and the alignment can therefore be used to extract sets of putative positional homologs [28]. Nucleotide regions in any given genome are aligned to any other genome only once. The 5282 gene sets are therefore restricted to single-copy gene families and range in size from 4 to 27 members. Using this approach, paralogous sequences are separated into distinct gene sets, thereby reducing the complications of paralogy for our LGT inference. Families of size *N *< 4 were excluded from further analysis as they do not contribute to meaningful phylogenetic inference; as a consequence, we are not able to identify within-clade LGT affecting families of size *N *< 4. Figure 1 shows the size distribution of the 5282 gene sets, and the representation of each of the 27 genomes among these gene sets is shown in Figure 2. The genome represented in the smallest number of the gene sets is that of the commensal *E. coli *Crooks strain (ATCC 8739). A number of pathogenic *E. coli *strains are also represented in relatively few of these gene sets: UMN026, APEC01, S88, UT189, ED1a and 536 are represented in fewer than 3350 families. With the exception of the group D strain UMN026, all these strains belong to phylogenetic group B2.

**Figure 3:**
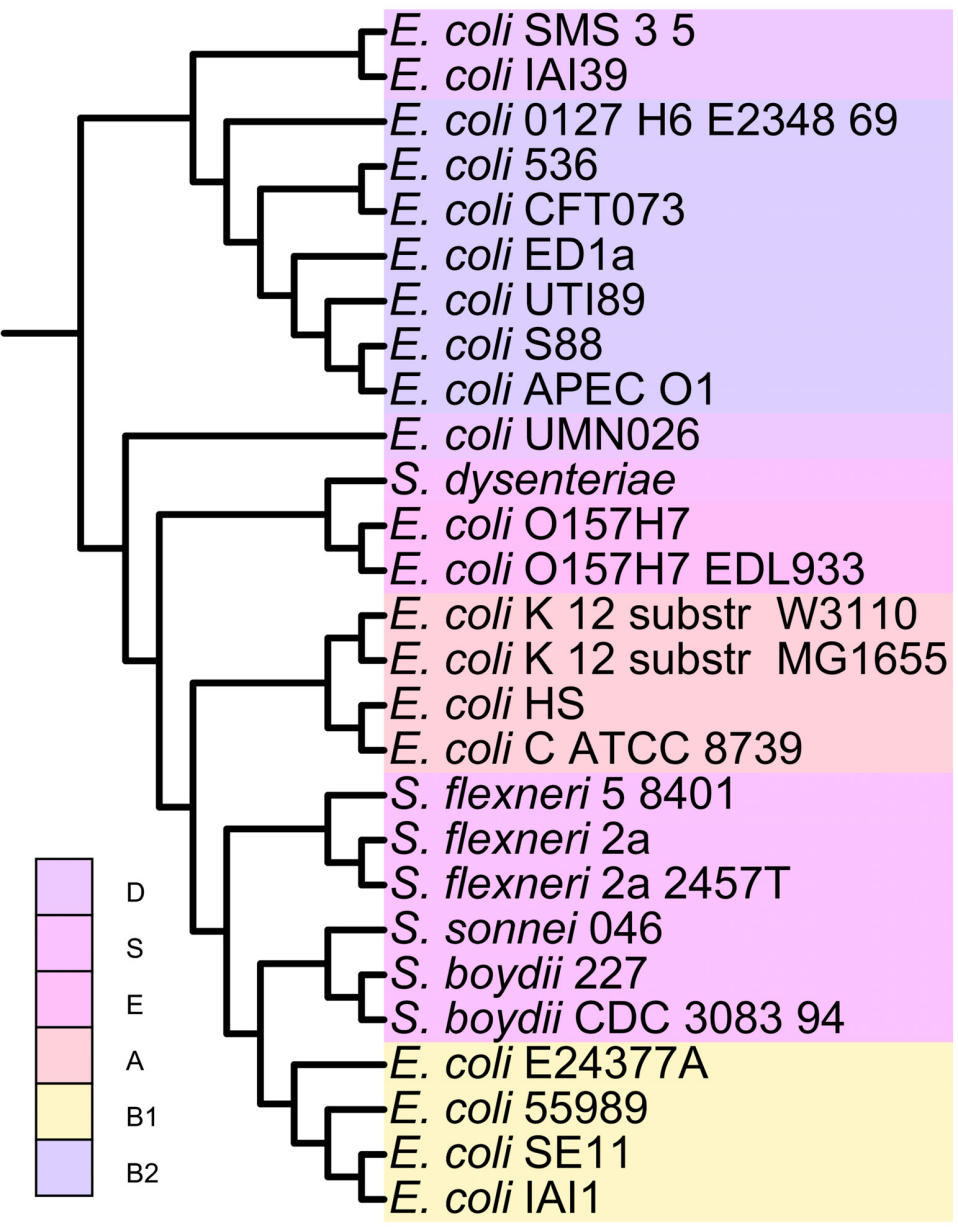
***E. coli - Shigella *reference tree**. Aggregate *E. coli - Shigella *reference tree reconstructed from 5282 Bayesian protein trees using matrix representation with parsimony (MRP) [31]. Colours indicate membership in *E. coli *phylogenetic groups.

We inferred Bayesian phylogenetic trees [29,30] separately for each of the 5282 *E. coli *protein sets and subsequently aggregated all adequately supported bipartitions (those with posterior probability (PP) ≥ 0.95) to generate an *E. coli - Shigella *reference tree using matrix representation with parsimony (MRP) [31]. Of the 287, 315 internal bipartitions in these 5282 individual protein trees, 113, 101 (39.4%) have posterior probability (PP) ≥ 0.95 and were used to compute the MRP tree. The resulting phylogeny (Figure 3) is our reference hypothesis about the vertical evolutionary relationships among the 27 *E. coli *and *Shigella*. We manually rooted the MRP tree based on the findings of Touchon *et al*. [32] who reconstructed the phylogenetic history of 20 *E. coli *and *Shigella *strains, all of which were included in our work, using *Escherichia fergusonii *as an outgroup.

**Figure 4:**
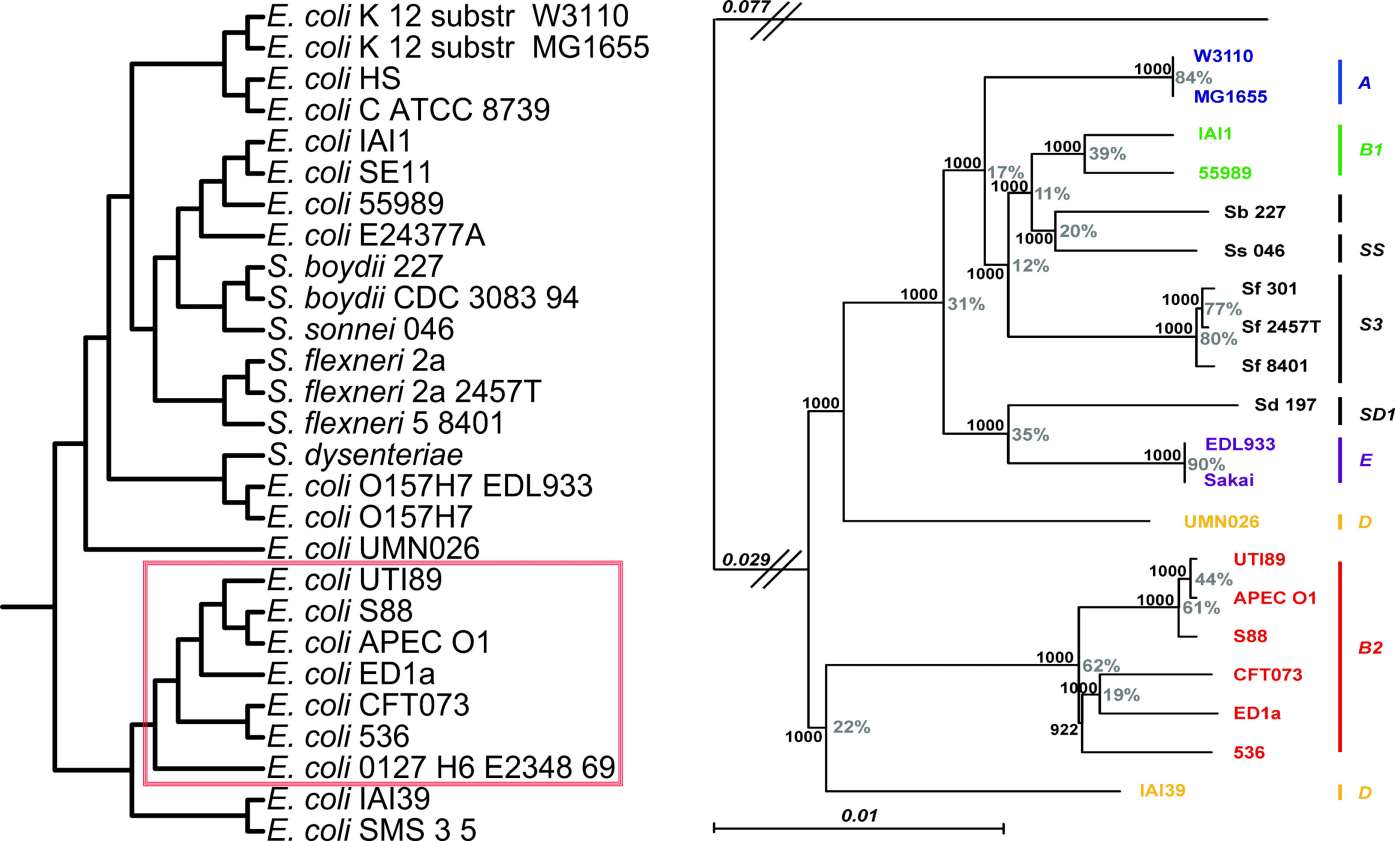
**MRP *E. coli *- *Shigella *reference tree and a previously reported *E. coli - Shigella *maximum likelihood core phylogeny**. Left: Aggregate MRP phylogenetic tree of 27 *E. coli *and *Shigella *strains reconstructed from 5282 Bayesian protein trees. Right: Maximum likelihood phylogenetic tree of 20 *E. coli *and *Shigella *strains reconstructed from the sequences of 1878 genes of the *Escherichia *core genome (taken directly from [32]) The B2 subtree, which contains a subset of bipartitions that are discordant between these two trees, is highlighted by a red box on the MRP tree.

Our MRP tree is remarkably concordant with the *E. coli - Shigella *phylogeny reported by Touchon *et al*. [32], which was inferred using a maximum likelihood approach based on 1878 concatenated *E. coli - Shigella *core gene sequences (Figure 4). Like the Touchon *et al*. tree, our MRP tree reconstructs four of the five ECOR phylogenetic groups as monophyletic, with only group D recovered as polyphyletic (Figure 4). Three group D strains are represented in our MRP tree: two (*E. coli *IAI39 and *E. coli *SMS-3-5) are recovered as closely related to the group B2 strains, while the third D strain (*E. coli *UMN026) forms a clade with *Shigella *and the strains belonging to phylogroups A, B1 and E. Only three bipartitions of our MRP tree are discordant with the phylogeny recovered by Touchon *et al*. [32], all of which occur within the B2 subtree (Figure 4).

Next, using a rigorous two-phase approach [33] we examined each of the 5282 aligned gene sets for evidence of genetic recombination. As the second phase of this approach is based on phylogenetic discrepancy, inference of an internal recombination breakpoint constitutes *prima facie *evidence of within-gene genetic transfer in a lineage leading to one or more of these genes. Following the classification system proposed by Chan *et al*. [15], we found clear evidence (class A, B or C) of recombination breakpoints in 678 gene sets (12.8% of 5282). We also compared each of the 5282 Bayesian protein trees to the MRP reference tree. Of the 5282 gene sets, 2440 (46.3%) were found to yield protein trees discordant with the MRP tree. These protein trees do not share the common phylogenetic signal in the MRP reference tree and are interpreted as providing putative evidence of LGT within the *E. coli *- *Shigella *lineage.

We found evidence for LGT in 2655 (50.3%) gene sets: 678 (12.8%) show evidence of one or more observable recombination breakpoints within the boundaries of the gene, while a further 1977 (37.4%) yield protein trees that are topologically discordant with the reference tree but do not contain one or more observable recombination breakpoints (Table 1). Using the terminology introduced by Chan *et al*. [16], we refer to these gene sets as observable recombination breakpoint positive (ORB+) and observable recombination breakpoint negative (ORB-) respectively. The latter represent putative instances of the lateral transfer of the entire open reading frame (or beyond) within the *E. coli *- *Shigella *lineage. Of the 678 gene sets that yielded observable recombination breakpoints, 215 were found to be not topologically discordant with the MRP tree.

**Table 1:** Lateral genetic transfer within the *E. coli - Shigella *clade.

Category	Observable recombination breakpoint(s)	No observable recombination breakpoint(s)	Totals
Protein tree discordant with MRP reference tree	463 Within-gene (fragmentary) lateral	1977 Whole-gene lateral	2440
Protein tree concordant with MRP reference tree	215 Within-gene (fragmentary) lateral	2627 Vertical	2842

Totals	678	4604	5282

**Figure 5:**
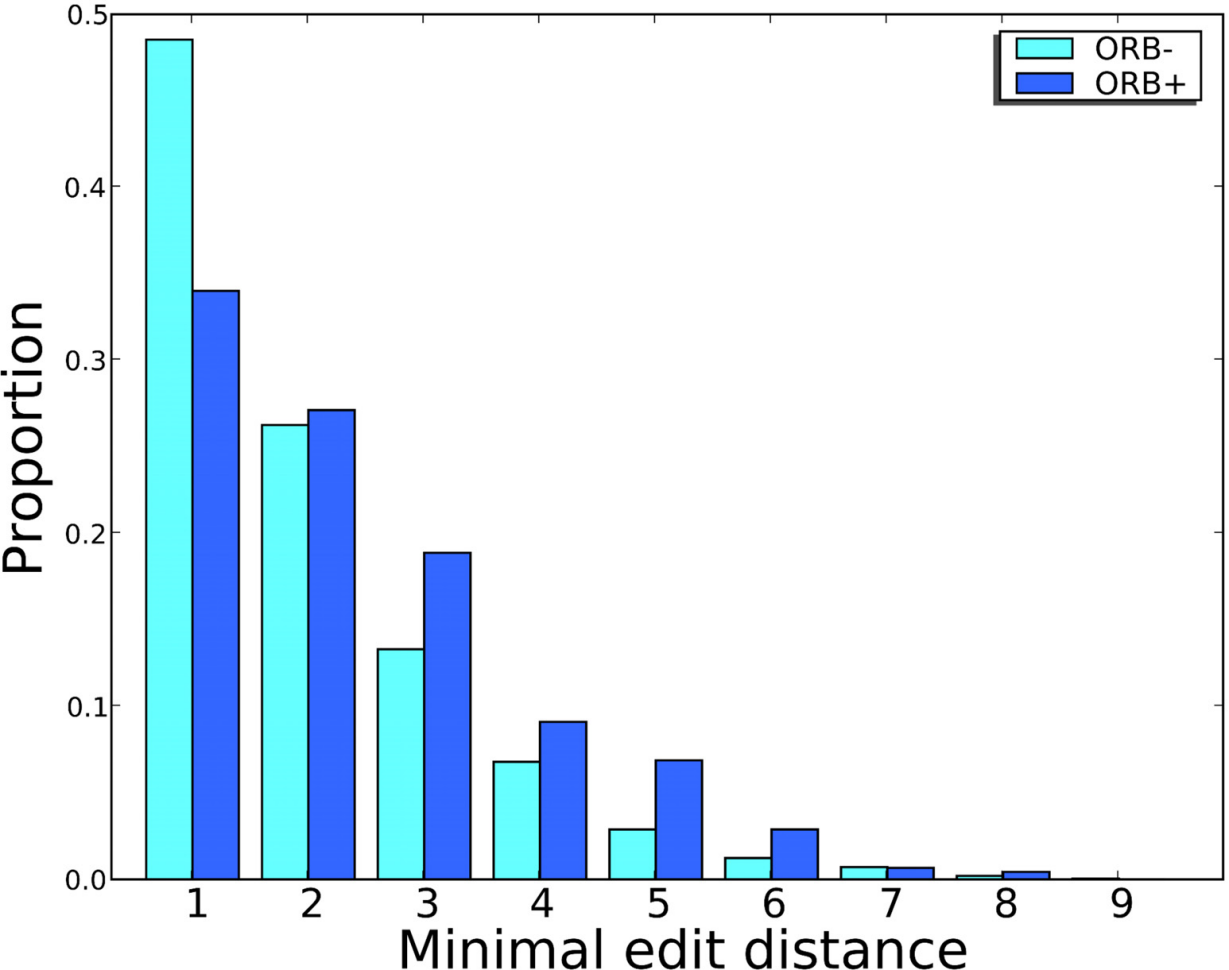
**Distribution of edit distances for 2389 *E. coli - Shigella *protein sets which yielded discordant protein trees**. ORB+ gene sets have a higher edit distance than ORB- gene sets (*P *< 0.001, by Wilcoxon rank sum test; 450 ORB+ gene sets and 1939 ORB- gene sets).

Subtree prune-and-regraft (SPR) operations can be applied to reconcile topological discordance between two phylogenetic trees. In an SPR operation, any edge of a binary tree T (i. e. a tree in which all non-leaf vertices have degree three) is cut, thereby giving two subtrees T' and T''. The subtree T'' is then regrafted by annealing the same cut edge to a new vertex in T' created by the annealing operation. Each SPR operation can be interpreted as equivalent to an LGT event involving a donor and recipient lineage. The regrafted edge corresponds to the donor taxon and the cut edge corresponds to the recipient taxon. Individual protein trees may be more or less discordant with the MRP reference topology, *i.e*. require different numbers of SPR operations on the reference tree to reconcile the observed discordance [6,34]. The minimum number of SPR operations required to reconcile observed discordance between a test tree and the reference tree is referred to as its *edit distance*. Unfortunately, computing the edit distance between two unrooted trees is a nondeterministic polynomial-time (NP)-hard problem [35], which in practice means that for sufficiently large data (here, the number of sequences related by the tree) it is impossible to know whether a globally optimal solution has been found. We were able to recover an edit distance for 2389 (98%) of the 2440 test trees. These distances range between 1 (1094 trees) and 9 (1 tree); however, more than 70% of the discordant test trees have an edit distance ≤ 2, indicative of two or fewer transfer events (Figure 5). Although the extent of discordance is variable across gene sets, instances of discordance implying more-complex patterns of LGT are in a clear minority. ORB+ gene sets have a higher edit distance than ORB- gene sets (*P *< 0.001, by Wilcoxon rank sum test; 450 ORB+ gene sets and 1939 ORB- gene sets).

Our results (summarised in Table 1) demonstrate that within-species LGT plays an important part in the evolution of *E. coli *- *Shigella *genomes. As in the 144-genome [16] and *Staphylococcus *studies [17], a solely whole-gene approach would have overlooked a major proportion of LGT. Our next goal was to use these LGT data to assess the contribution of within-species genetic exchange to TRN network evolution.

### Lateral genetic transfer and the transcriptional regulatory network

A list of *E. coli *K12 genes encoding TFs, and the corresponding target genes, were extracted from RegulonDB [36] and used to construct a *E. coli *K12 TRN. In this network, the genes are represented as nodes and regulatory interactions as directed edges. The network has 1577 nodes and 3804 edges which encompass 179 unique TF-encoding genes enacting regulation of 1533 target genes; as TF-encoding genes are themselves targets of other TFs, 'target genes' and 'TFs' are not mutually exclusive categories.

Among the 179 TF-encoding genes in the reconstructed TRN, 85 (47.5%) were identified as lateral and another 85 (47.5%) as vertical. A further nine remained unclassified, as it was not possible to carry out phylogenetic analysis on the corresponding gene sets (see Methods). We calculated the out-degree and betweenness of the TF-encoding nodes to assess whether LGT and non-LGT nodes occupy different positions in the TRN. Out-degree is the number of target genes regulated by a given TF node, and betweenness measures the frequency at which a given node lies on the shortest path between any pair of nodes in the network [37]. We found that vertical TF nodes have higher out-degree than lateral TF nodes (*P *= 0.001) and also have higher betweenness (*P *= 0.04, both by Wilcoxon rank sum test). Thus, vertical TF nodes regulate larger numbers of target genes and are found at more-central positions in the TRN than are lateral TF nodes. This suggests that high centrality may be a barrier to intra-clade transfer of TF genes; however, these results need to be interpreted cautiously, as the out-degree distribution for TF nodes is skewed.

The out-degrees of the nodes of TRNs have previously been shown to follow a scale-free distribution, characterized by a small proportion of highly connected *hub *nodes and a relative large proportion of weakly connected nodes [38]. For example, in the reconstructed *E. coli *K12 TRN, the 20 TFs that regulate the largest number of genes enact 2617 (68.8%) of the 3804 regulatory interactions represented in the network. Given the large influence of so few regulators, we have followed the classification system used by Price *et al*. [14] to assign regulatory genes into two major categories on the basis of their corresponding number of target genes: *global regulators*, which comprise the 20 TFs that regulate the largest number of genes, and *neighbour regulators*, which regulate adjacent genes in the genome. Eight TF genes which regulate adjacent genes were excluded from the list of neighbour regulators because they are global regulators. Of the 179 unique regulatory genes represented in the network, 22 were assigned as global regulators and 93 as neighbour regulators. A further 64 regulatory genes fell within neither of these two categories and were treated as a third category which we refer to as *other (non-global) regulators*. In the following sections, we examine the evolutionary histories of regulatory genes in each of these three categories separately to determine if LGT has affected these different modes of regulation differently.

### Most global regulators evolve vertically within the *E. coli *- *Shigella *clade

We found that gene sets coding for global regulators are less likely than other protein-coding genes to yield discordant protein trees (*P *= 0.001) and are also less likely to be ORB+ (*P *= 0.048, both by Fisher's exact test). Among the 22 global regulatory gene sets (two of the 20 global TFs examined are active as heterodimers), only three were found to yield protein trees that are discordant with our MRP reference tree (Table 2), and none showed evidence of one or more internal recombination breakpoints. This indicates that global regulators have mostly evolved vertically within the *E. coli - Shigella *clade.

**Table 2:** Lateral genetic transfer of global regulators within the *E. coli - Shigella *clade.

TF name	Gene coding for the TF	Blattner number	Number of target genes	Is the corresponding protein tree concordant or discordant with the *E. coli - Shigella *MRP tree?	Evidence for internal recombination breakpoint(s)?
CRP	*crp*	b3357	435	Concordant	No
FNR	*fnr*	b1334	282	Concordant	No
Fis	*fis*	b3261	225	Concordant	No
IHF	*ihfA*	b1712	217	Concordant	No
IHF	*ihfB*	b0912	217	Discordant	No
ArcA	*arcA*	b4401	158	Concordant	No
H-NS	*hns*	b1237	144	Concordant	No
NarL	*narL*	b1221	114	Concordant	No
Lrp	*lrp*	b0889	97	Concordant	No
Fur	*fur*	b0683	85	Concordant	No
nsrR	*nsrR*	b4178	84	Concordant	No
FlhDC	*flhD*	b1892	79	Concordant	No
FlhDC	*flhC*	b1891	79	Concordant	No
CpxR	*cpxR*	b3912	56	Concordant	No
LexA	*lexA*	b4043	55	Concordant	No
NarP	*narP*	b2193	49	Concordant	No
ModE	*modE*	b0761	47	Discordant	No
NtrC	*glnG*	b3868	44	Discordant	No
FruR	*fruR*	b0080	40	Concordant	No
ArgR	*argR*	b3237	38	Concordant	No
PhoP	*phoP*	b1130	37	Concordant	No
PhoB	*phoB*	b0399	37	Concordant	No

Global regulators are the 20 TFs that regulate the largest number of genes in the *E. coli *K12 TRN.

Here we have assigned regulators as global on the basis of their corresponding number of target genes, but this definition excludes TFs that are encoded by genes that are less well-connected in the network but are nonetheless important for cell fitness. Martínez-Antonio and Collado-Vides [2] have identified an alternative set of *E. coli *global TFs based on expanded criteria which included, among others, the variety of conditions under which the regulator exerts control. Seven TFs in *E. coli *satisfy all global TF criteria outlined by Martínez-Antonio and Collado-Vides [2] (CRP, FNR, ArcA, LRP, FIS, IHF and H-NS). A second tier consisting of nine *E. coli *regulators was also identified that, while less-connected in the network, were considered important for cell fitness (NarL, Fur, CpxR, PhoB, PurR, Rob, DgsA (formerly Mlc), CspA, SoxR). This set of regulators includes five TFs (PurR, Rob, DgsA (formerly Mlc), CspA, SoxR) that were not included in the set of global regulators we identified on the basis of their number of target genes. Among all 16 *E. coli *global TFs identified by Martínez-Antonio and Collado-Vides [2], only three yield protein trees that are discordant with MRP reference tree, and none shows evidence of one or more internal recombination breakpoints (Table 3). Thus, even under an alternative definition of 'global', these regulators have mostly evolved vertically and are not subject to within-gene transfer. These findings confirm and extend a previous report that in *E. coli *global regulators are less susceptible to LGT than other regulatory genes [14].

**Table 3:** Lateral genetic transfer of global regulators within the *E. coli *- *Shigella *clade using an alternative definition of 'global regulator'.

TF name	Gene coding for the TF	Blattner number	Number of target genes	Is the corresponding protein tree concordant or discordant with the *E. coli - Shigella *MRP tree?	Evidence for internal recombination breakpoint(s)?
CRP	*crp*	b3357	435	Concordant	No
FNR	*fnr*	b1334	282	Concordant	No
Fis	*fis*	b3261	225	Concordant	No
IHF	*ihfA*	b1712	217	Concordant	No
IHF	*ihfB*	b0912	217	Discordant	No
ArcA	*arcA*	b4401	158	Concordant	No
H-NS	*hns*	b1237	144	Concordant	No
Lrp	*lrp*	b0889	97	Concordant	No
NarL	*narL*	b1221	114	Concordant	No
Fur	*fur*	b0683	85	Concordant	No
CpxR	*cpxR*	b3912	56	Concordant	No
PhoB	*phoB*	b0399	37	Concordant	No
PurR	*purR*	b1658	31	Concordant	No
Rob	*rob*	b4396	18	Concordant	No
DgsA (Mlc)	*dgsA*	b1594	10	Discordant	No
CspA	*cspA*	b3556	3	Concordant	No
SoxR	*soxR*	b4063	3	Discordant	No

Definition of a global regulator as given in [2].

### Neighbour regulators have frequently been transferred both intact and as within-gene fragments between strains of *E. coli *and *Shigella*

In contrast to global regulators (which, as we have just seen, have mostly evolved vertically within the *E. coli - Shigella *clade), we found evidence for LGT in at least 56 (62%) of the 90 neighbour regulator gene sets of size *N *≥ 4: 19 (34%) of these were found to be ORB+, while a further 37 (41%) yielded protein trees that are topologically discordant with the reference tree but are ORB- (Table 4). These sets of neighbour regulator genes are more likely than other protein-coding genes to have experienced within-clade LGT (*P *= 0.01, by Fisher's exact test). The proportion of neighbour regulators that yield discordant protein trees (49 of 90, 54%) is comparable to the proportion for all protein-coding genes (2440 of 5282, 46.2%) (the proportions are not significantly different: *P *= 0.1, by Fisher's exact test); however, neighbour regulator gene sets are more likely to be ORB+ than are other protein-coding genes (*P *= 0.02, by Fisher's exact test).

**Table 4:** Lateral genetic transfer of neighbour regulators within the *E. coli *- *Shigella *clade.

Category	Observable recombination breakpoint(s)	No observable recombination breakpoint(s)	Totals
Protein tree discordant with MRP reference tree	12 Within-gene (fragmentary) lateral	37 Whole-gene lateral	49
Protein tree concordant with MRP reference tree	7 Within-gene (fragmentary) lateral	34 Vertical	41

Totals	19	71	90

**Figure 6:**
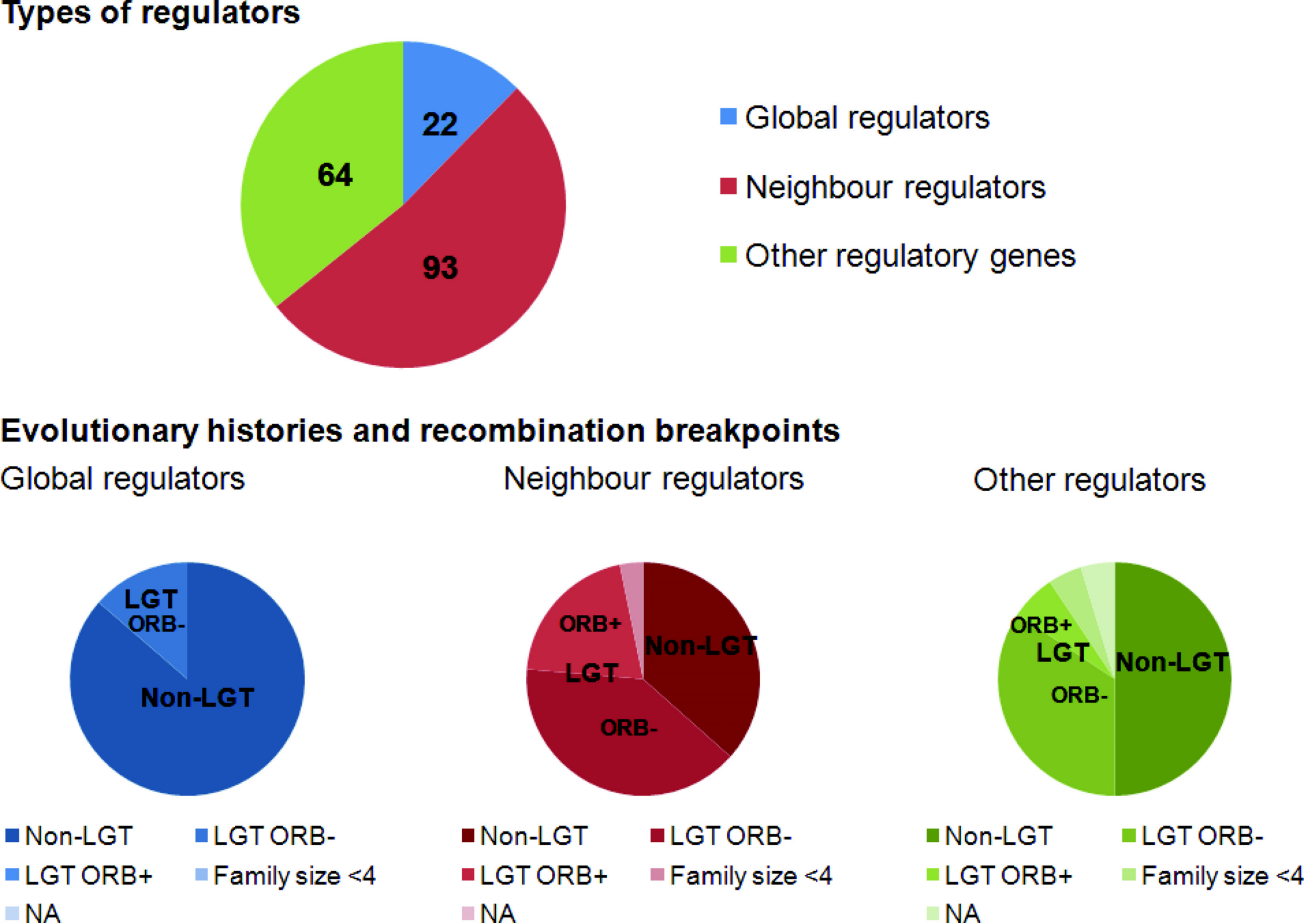
**Lateral transfer of TF genes within the *E. coli - Shigella *clade**. TFs were separated into three main groups: global regulators, neighbour regulators and other regulators. TF-encoding gene sets that yielded proteins trees that were discordant with the MRP reference tree and/or were ORB+ were classified as LGT, whereas gene sets that yielded concordant protein trees and had no recombination breakpoints within the gene boundaries were classified as non-LGT. The LGT genes were further divided into two groups: ORB+ and ORB-.

ORB- instances of putative LGT involving neighbour regulator gene sets are almost twice as common as ORB+ instances. Thus the incorporation of intact neighbour regulators is more frequent than recombination involving within-gene fragments. Neighbour regulator gene sets were more likely to show evidence of LGT than other non-global regulatory gene sets (Table 5) (*P *= 0.03) and were also more likely to be ORB+ (*P *= 0.02, both by Fisher's exact test). The proportion of neighbour regulator gene sets that yield discordant protein trees (49 of 90, 54%) is comparable to that of other non-global regulators (24 of 58, 41%) (the proportions are not significantly different: *P *= 0.1, by Fisher's exact test). LGT of global, neighbour and other non-global regulators is summarised in Figure 6.

**Table 5:** Lateral genetic transfer of other regulators within the *E. coli *- *Shigella *clade.

Category	Observable recombination breakpoint(s)	No observable recombination breakpoint(s)	Totals
Protein tree (amino acid) discordant with reference	2 Within-gene (fragmentary) lateral	22 Whole-gene lateral	24
Protein tree (amino acid) concordant with reference	2 Within-gene (fragmentary) lateral	32 Vertical	34

Totals	4	54	58

Other regulators are the regulatory gene sets which fall within neither the neighbour or global regulator categories.

**Figure 7:**
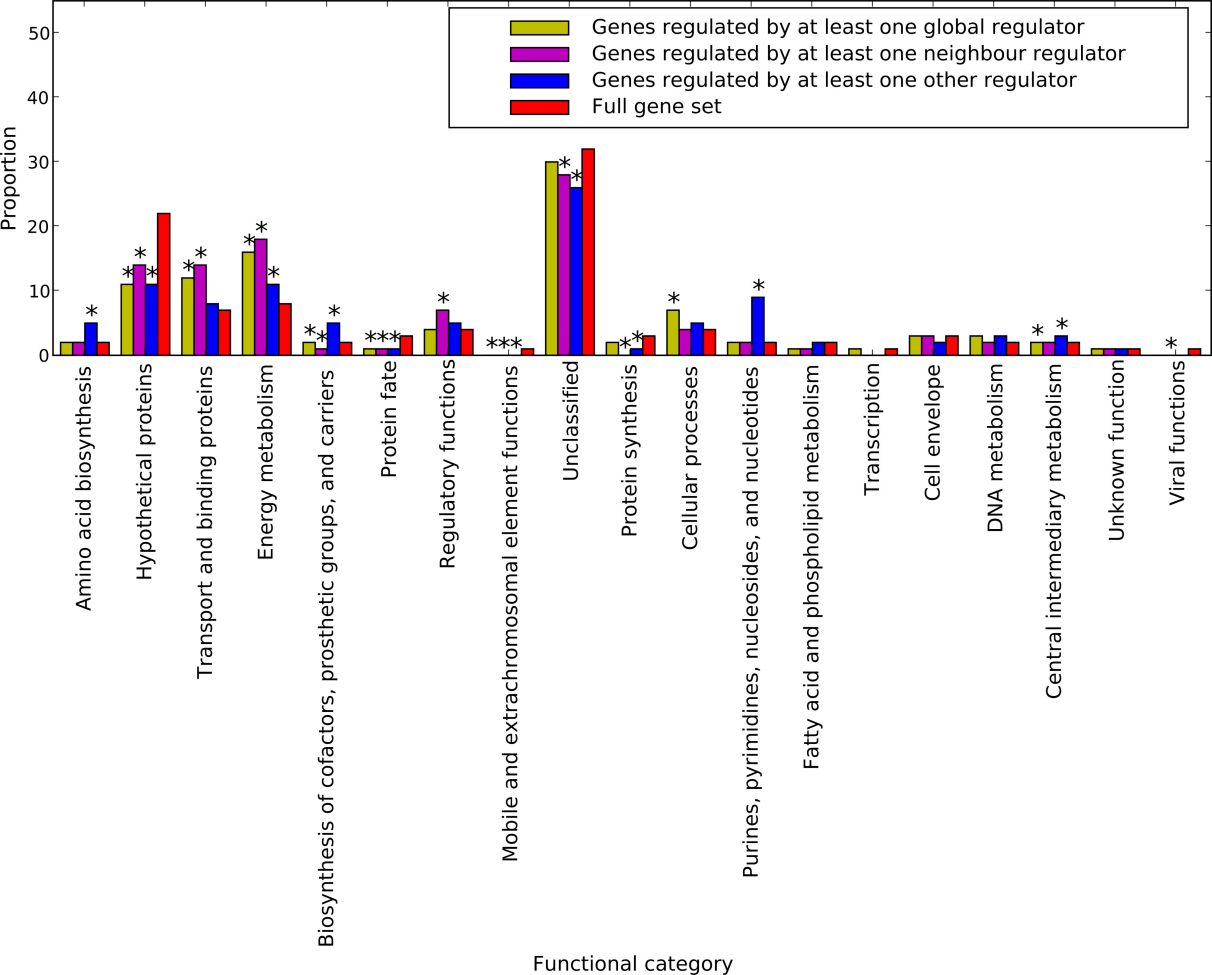
**Distribution of genes among JCVI functional categories for *E. coli *K12 genes**. Shown for each functional category are proportions of genes regulated by global regulators, by neighbour regulators, and by other non-global regulators. Those significantly over- or underrepresented (*P *< 0.05, by Fisher's exact test) vis-a-vis their frequency in the full set of *E. coli *K12 genes are marked with an asterisk.

Function of the gene product (here, a protein) is correlated with relative susceptibility, or resistance, to LGT. In particular, genes involved in DNA replication, transcription and translation (informational genes) have been shown to be less susceptible to transfer than are genes participating in housekeeping functions such as controlling energy metabolism and the biosynthesis of nucleotides and amino acids (operational genes) [39,40]. To examine possible functional bias pertaining to the high frequency of LGT among neighbour regulator protein sets, we used annotations from The J. Craig Venter Institute (JCVI) Comprehensive Microbial Resource http://cmr.jcvi.org/ to assign a functional category to all protein-coding genes in *E. coli *K12. We then compared the functions of genes regulated by global, neighbour and other (non-global) regulators. Figure 7 shows, for each functional category, the proportion of genes regulated by each type of regulator (global, neighbour and other) compared to its frequency among the full set of 4149 *E. coli *K12 protein-coding genes.

Genes regulated by at least one neighbour regulatory gene are significantly over- or underrepresented in ten (more than half) of the JCVI functional categories (*Hypothetical proteins*; *Transport and binding proteins*; *Energy metabolism*; *Biosynthesis of cofactors*, *Prosthetic groups, and carriers*; *Protein fate*; *Regulatory functions*; *Mobile and extrachromosomal element functions*; *Unclassified*; *Protein synthesis*; and *Viral functions*). Four of these ten functional categories are correspondingly over- or underrepresented for the sets of genes regulated by global and other (non-global) regulators (*Hypothetical proteins*; *Energy Metabolism*; *Protein fate*; and *Mobile and extrachromosomal element functions*). We did not observe any functional categories to be overrepresented for genes regulated by neighbour regulators but underrepresented in genes regulated by global or other regulators. *Biosynthesis of cofactors, prosthetic groups, and carriers *is the only functional category underrepresented for genes regulated by neighbour regulators but overrepresented for genes regulated by other regulators; however, given the relatively small proportion of *E. coli *K12 genes that fall within this category it is not likely to be a significant source of functional bias. These results suggest that the differences between the transfer frequencies of global, neighbour and other (non-global) regulatory genes are not due to the function of the genes they regulate.

### Neighbour regulators and their target genes evolve independently via LGT within the *E. coli *- *Shigella *clade

**Figure 8:**
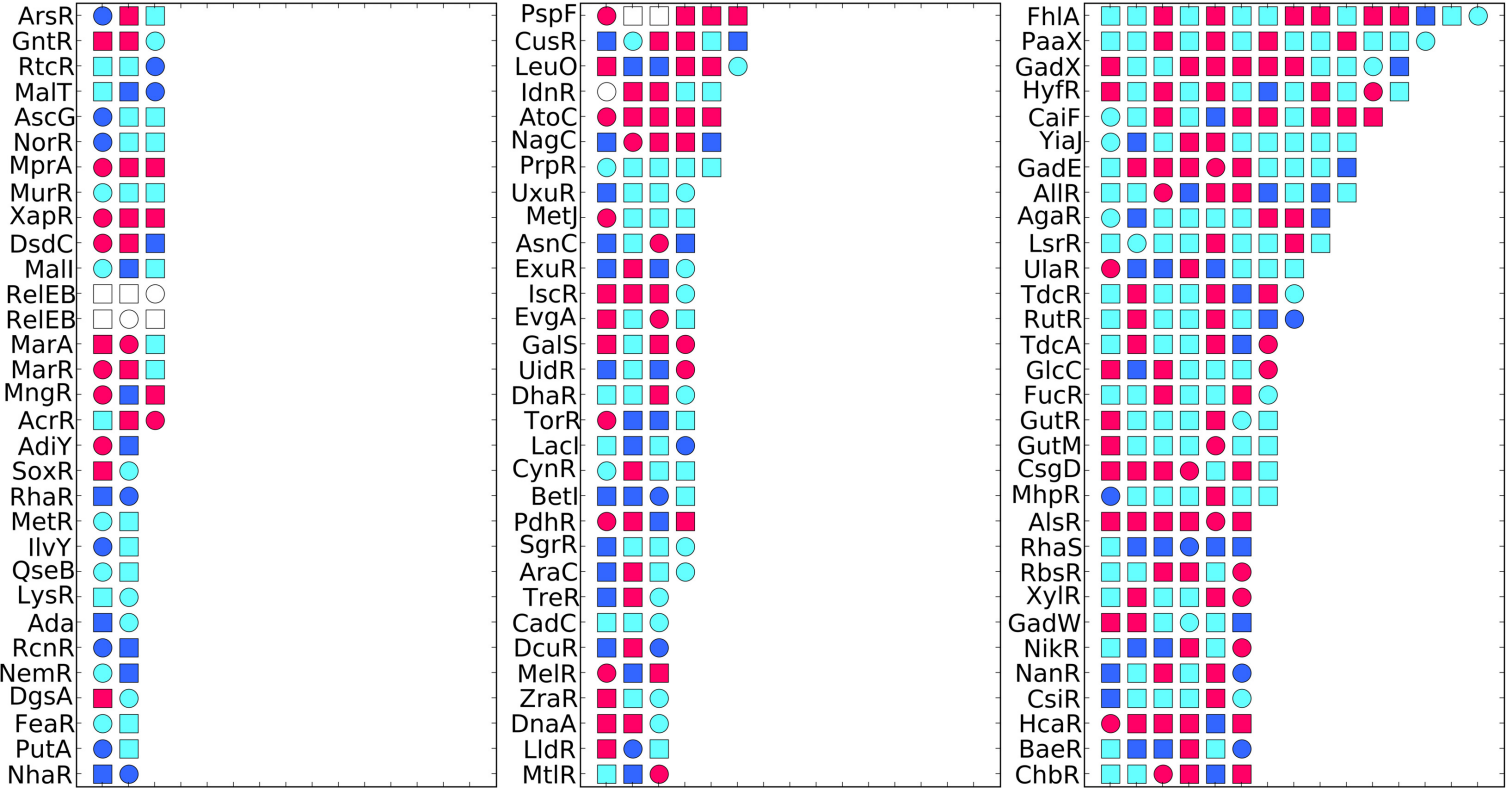
**Evolutionary histories of neighbour regulators and their nearby target genes**. Shown here is a schematic representation of 93 regulatory neighbourhoods. Genes encoding TFs are represented as circles and the corresponding nearby target genes of the TF as squares. Genes are coloured accordingly: non-LGT genes are red, ORB+ genes are dark blue, ORB- genes light blue and genes for which LGT information is not available are white. 'Non-LGT' refers to gene sets that yielded a phylogeny that was concordant with the *E. coli - Shigella *reference tree and had no recombination breakpoints within the gene boundaries.

We next examined the phylogenetic histories of neighbour regulators and their nearby target genes to determine if there is a propensity toward co-transfer of the elements involved in neighbour regulatory interactions (Figure 8). We introduce the term *regulatory neighbourhood *to describe the set of adjacent target genes whose expression is directly modulated by a given neighbour regulator. Each regulatory neighbourhood encodes a set of collinear genes which includes a TF-encoding gene that regulates all other genes in the neighbourhood. TF genes may be upstream or downstream of their regulatory targets. We consider the neighbour regulator itself part of the regulatory neighbourhood even if autoregulation is not present. The number of regulated genes in the regulatory neighbourhoods investigated here varies widely. While many TF-encoding genes regulate only one adjacent target gene, others regulate many: the gene encoding TF FhlA, for example, regulates 14 collinear target genes adjacent on the *E. coli *K12 chromosome (Figure 8).

If a neighbour regulator and its nearby target genes have been co-transferred within the *E. coli - Shigella *lineage, we would expect that each of the gene sets in the regulatory neighbourhood will yield protein trees that are topologically discordant with the MRP reference tree and that support the same evolutionary history of *E. coli *and *Shigella *strains. In the case of recent co-transfer we would expect all genes in the neighbourhood to be ORB-. Alternatively, a more-ancestral co-transfer could be followed by successive within-region LGT events which over time superimpose DNA onto the previously incorporated region. In such cases, we would expect regulatory neighbourhoods to be frequently interrupted by internal recombination breakpoints.

In total, there are 93 neighbour regulator gene sets corresponding to 90 unique regulatory neighbourhoods (three regulatory neighbourhoods encode more than one TF which regulates the same set of nearby target genes). Among the 37 neighbour regulators found to be ORB-, only seven belong to regulatory neighbourhoods composed exclusively of ORB- genes; of these seven, none contains gene sets that all support the same (protein) evolutionary history. Moreover, 56 (62%) of these 90 regulatory neighbourhoods contain at least one observable recombination breakpoint. This suggests that regulatory neighbourhoods are probably not co-transferred as genomic stretches of collinear intact genes, or if they are, co-transfer has often been followed by successive layering of LGT events within the region. One possible scenario is LGT of the regulatory neighbourhood into the *E. coli - Shigella *lineage from an external clade, followed by transfer of within-neighbourhood gene fragments within the lineage. Only four of the 93 neighbourhoods investigated show no evidence of transfer within the *E. coli - Shigella *lineage, suggesting that LGT has been very important in the evolution of these regions.

Price *et al*. [14] reported that in *E. coli*, many neighbour regulatory genes have been acquired by LGT, often simultaneously with the gene(s) they regulate. In contrast, we found no evidence to suggest that genes participating in neighbour regulatory interactions are constrained to co-transfer within the *E. coli - Shigella*. These authors used genes as the unit of analysis; here we find 19 (21%) of 90 neighbour regulator gene sets examined to be ORB+. Our finding that regulatory neighbourhoods have been frequently interrupted by internal recombination breakpoints suggests the DNA has been successively superimposed onto these regions. Such layering of LGT events may defy analysis, as signatures of ancestral co-transfer of neighbour regulators and their target genes have been overwritten. Our findings extend an earlier report that bacterial TFs do not usually co-evolve with their regulated genes [41].

### Regulation of LGT genes is no more complex than regulation of non-LGT genes

**Figure 9:**
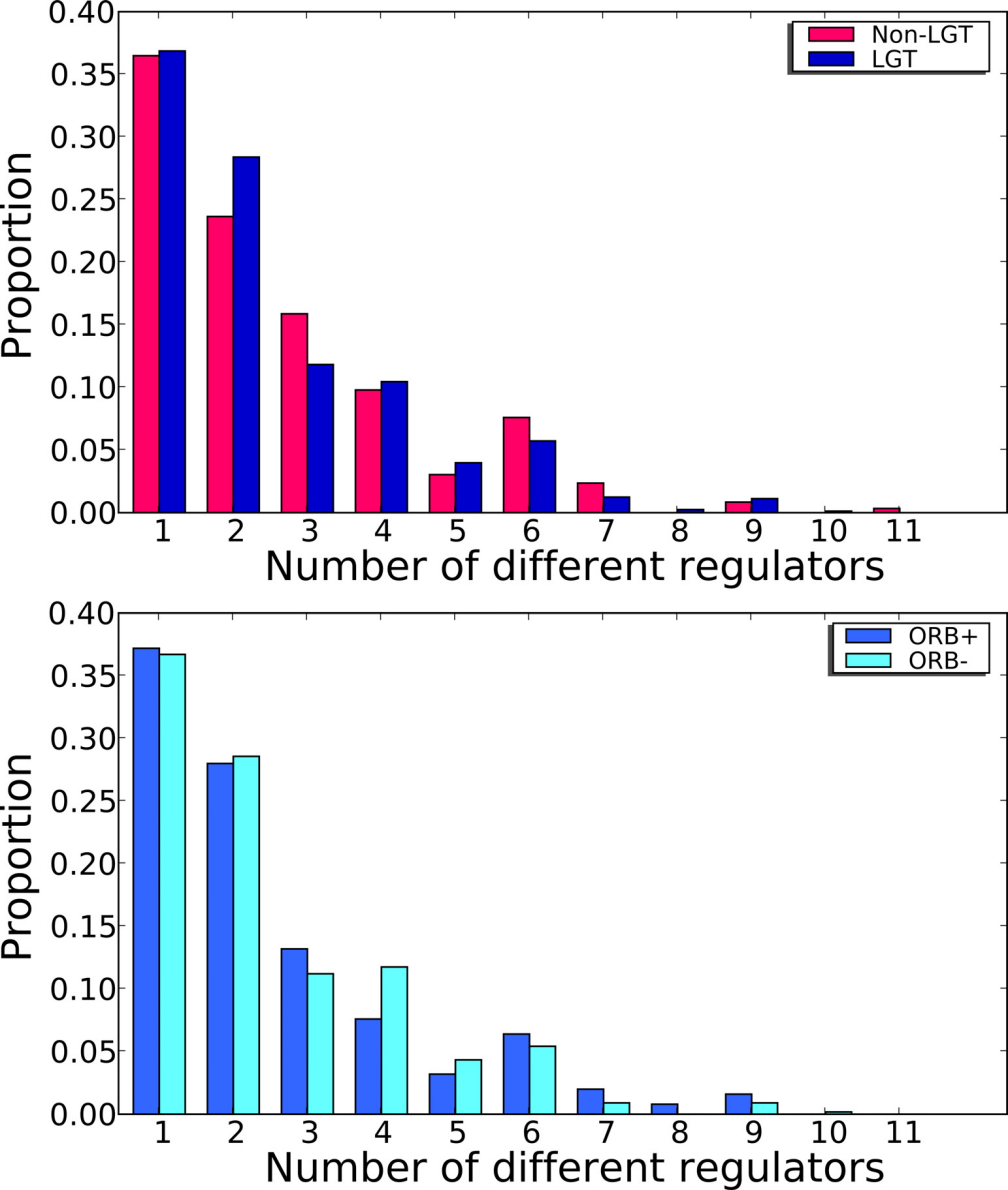
**Regulation of genes which have been laterally transferred within the *E. coli *- *Shigella *clade**. Genes without regulation and genes belonging to families of size *N *< 4 are not included. Top: 'Non-LGT' refers to gene sets that yielded a phylogeny that was concordant with the *E. coli - Shigella *reference tree and had no recombination breakpoints within the gene boundaries. 'LGT' refers to gene sets that were found to be ORB+ and/or yielded a protein tree that was discordant with the reference tree. LGT genes show no evidence of having more regulators than non-LGT genes (*P *= 0.3, by Wilcoxon rank sum test; 803 LGT genes and 592 non-LGT genes). Bottom: ORB- genes and ORB+ genes show no difference in the number of genes they are regulated by (*P *= 0.96, by Wilcoxon rank sum test; 250 ORB+ genes and 553 ORB- genes).

Network representations of transcriptional regulation have previously revealed that, on average, each target gene is regulated by two TFs [42-44]. We examined the regulation of LGT genes to determine if they are more or less likely than other genes to be regulated by multiple TFs. We found genes transferred within the *E. coli - Shigella *clade, and genes which have evolved vertically within the lineage, have a comparable number of regulators (*P *= 0.3, by the Wilcoxon rank-sum test) (Figure 9). This suggests that, at least in this case, genetic transfer among closely related strains is not constrained by relative complexity of regulation. This result is in contrast to a previous report by Price *et al*. [14] that genes of lateral origin exhibit more-complex regulation (tend to be regulated by more regulators) than non-LGT genes. The authors hypothesized that many LGT genes are niche-specific and therefore require more-complex regulation.

To account for these contrasting results, we note a number of differences between the Price *et al*. study and ours. Price *et al*. [14] focused on the evolutionary period since the divergence of *E. coli *from *Shewanella*, and included lateral transfers into the lineage from external clades, whereas we focus only on transfers within *E. coli *and *Shigella*. As mentioned above, Price *et al*. [14] took whole genes as their units as analysis for LGT identification. We examined the regulation of ORB+ genes to determine if they are any more or less likely than genes which have been transferred intact to be regulated by multiple regulators, and found no difference in the relative complexity of regulation of ORB+ and ORB- lateral gene sets (*P *= 0.96, by the Wilcoxon rank sum test) (Figure 9).

## Discussion

Gene regulation at the level of transcriptional control involves a complex network of regulatory interactions, one aspect of which is clearly demonstrated by the combinatorial control of individual genes by multiple TFs (Figure 9). How lateral genes connect into such complex networks has remained an open issue [18], although some general network properties of laterally transferred genes have been identified by intersecting public network data with lists of genes detected as lateral based on methods which assume genes are transferred as intact units [13,14,45].

Here we inferred 678 (12.8%) of 5282 *E. coli - Shigella *orthologous protein-coding gene sets to be ORB+: 215 (31.7%) of these yielded protein trees that were concordant with the *E. coli - Shigella *MRP tree. Thus, had we carried out our phylogenetic analysis of LGT based on entire genes only, we would have missed more than 30% of the lateral signal introduced by within-gene LGT and more than 8% (215 of 2655 lateral gene sets) of all lateral signal within the clade. The importance of accounting for within-gene transfer is even more-striking in the subset of protein-coding *E. coli - Shigella *gene sets that code for TFs, where almost 40% of ORB+ gene sets yield a protein tree concordant with the reference tree. Among the 170 TF genes for which we could construct a phylogenetic tree, 23 were inferred to be ORB+, of which 9 (39%) were recovered as topologically concordant with the reference. Clearly, a complete picture of how LGT genes connect into TRNs requires consideration of the impact of both whole- and within-gene transfer on individual regulators and their target genes.

Although detection of recombination breakpoints within gene sets has provided a more-comprehensive view of the lateral signal in *E. coli - Shigella*, certain caveats are in order. Foremost, we identified LGT only among lineages of *E. coli *and *Shigella*, i.e. our analysis does not identify foreign genes or gene fragments acquired laterally from outside the clade. Second, we did not attempt to identify donor or recipient lineages for genes identified as whole- or within-gene lateral; here, this would have required the identification of all possible SPR operations that resolve observed discordance between a discordant protein tree and the MRP reference tree. In many cases, multiple unique minimum SPR paths are possible and the actual historical one cannot be determined [6,34]. It is even more complicated to match donor and recipient lineages where there has been successive layering of LGT events, as seems likely where we infer multiple recombination breakpoints within a gene set or neighbourhood.

Global and neighbour regulators play distinct regulatory roles. Our results confirm that these different roles imply specific constraints on the evolvability of these regulators. Most global regulators were found to evolve vertically. In the rare case that a global regulator exhibited evidence of transfer within the *E. coli - Shigella *clade, it appeared to have been transferred intact. In contrast, 56 (62%) of the 90 neighbour regulator gene sets for which we could construct phylogenetic trees showed evidence of LGT, and 19 (34%) of these 56 showed evidence of within-gene transfer. It is apparent that these different modes of regulation (i.e. global versus neighbour regulation) evolve in a very different manner, at least with respect to lateral transfers within the *E. coli - Shigella *clade.

If laterally acquired genetic material is to avoid being silenced and become fixed in its new host, its expression must be appropriately regulated and the gene products it encodes must interact successfully with host systems. In the case of genes encoding proteins which act as regulators, this includes establishing regulation of appropriate targets. It is striking that neighbour regulators have undergone more-frequent lateral genetic transfer than other non-global regulators. This suggests that lateral regulators that have introgressed near their target genes have a higher probability to become fixed within the recipient. A possible explanation is that physical proximity to target genes, in the immediate term, provides an advantage for the establishment of regulatory interactions following introgression of lateral regulators.

Co-transfer of TF genes with their targets might alleviate at least some of the difficulties associated with integration into TRNs, as this would provide a means for efficient transcriptional regulation in the short term following introgression [18]; however, we found no evidence that neighbour regulators are transferred with their nearby target genes. In fact, neighbour regulators and their target genes appear to evolve independently via LGT within the *E. coli - Shigella *clade. Regulatory neighbourhoods are frequently interrupted by internal recombination breakpoints, and consist of genomic regions with conflicting evolutionary histories. This is most readily explained by these regions having been constructed through successive superimposed LGT events which overwrite more-ancestral lateral signal and thereby complicate, or even defy, analysis. By the same token, we cannot rule out the possibility that ancestral co-transfers have been overwritten by subsequent LGT within regulatory neighbourhoods.

We found no difference in the relative complexity of regulation (i.e. the number of regulators) of lateral versus vertical genes: relative complexity of regulation does not appear to be a barrier to lateral transfer within the *E. coli - Shigella *clade. Although homologous replacement of a gene fragment, rather than transfer of an intact gene, might avoid some of the problems associated with recruitment of transcriptional regulators following introgression into the recipient genome [18], we found no evidence to suggest a difference in the relative complexity of regulation (i.e. number of TF regulators) of whole-gene LGT genes and within-gene LGT genes. These results are important because they suggest that relative complexity of regulation does not govern the impact of within-species LGT on TRNs.

## Conclusions

Our results demonstrate that the transfer of both intact genes and within-gene fragments has been frequent within the *E. coli - Shigella *clade. As more than 30% of ORB+ gene sets do not yield discordant trees, the frequency of LGT within the clade would have been significantly underestimated by a phylogenetic study that assumed intact genes as the unit of genetic transfer. We assessed the relative contribution of within- and whole- gene transfer to the evolution of individual regulators and their target genes as a necessary step toward understanding how lateral genes connect into TRNs and contribute to overall TRN evolution. It is clear that a substantial proportion of the individual gene components of TRNs have been subject to LGT within the *E. coli - Shigella *clade, and that different modes of regulation are impacted by LGT differently. We found that global regulators have mostly evolved vertically, and neighbour regulators have frequently been subject to genetic transfer within the *E. coli - Shigella *clade. Comparing genetic transfer in neighbour regulator and other non-global regulator genes sets, we observed a lower frequency in the latter. Given these findings, future work is well positioned to extend the questions asked here to consider how the lateral transfer of genetic material among more distantly related organisms contributes to TRN evolution and if this differs from the impact of LGT among strains of the same species.

## Methods

### Inference of gene sets

Twenty-seven completely sequenced *E. coli *and *Shigella *genomes were downloaded from the NCBI ftp server http://www.ncbi.nlm.nih.gov/Ftp/. A whole-genome alignment of the complete *E. coli *and *Shigella *genomes and four additional draft genomes (*E. coli *101-1, *E. coli *F11, *E. coli *O157H7 str EC440 and *Shigella *sp D9) was performed using the progressiveMauve algorithm of the MAUVE program version 2.3.0 [27] with default parameter settings. We then extracted sets of positionally homologous protein-coding genes for the 27 complete genome sequences from the alignment using the MAUVE 'export orthologs' function. The draft genomes were included in the initial alignment to provide better representation of the strains across the species; however, we did not include sequences from these genomes in our sets of positional homologs as annotation of these genomes was incomplete and we wanted to only include annotated protein-coding genes. In total, 5282 sets of putatively orthologous protein-coding genes of size *N *≥ 4 were inferred. We refer to these sets interchangeably as protein or gene sets if one or the other term is not required by context.

### Alignment and phylogenetic tree construction

The amino acid sequences corresponding to the 5282 protein sets (families) of size *N *≥ 4 were extracted from GenBank and aligned using ProbCons [46] with default parameter settings. Following alignment, ambiguously aligned regions of the alignments were removed using GBLOCKS version 0.91b [47] with the following parameter settings: minimum number of sequences for a conserved position (*n*/2)+1; minimum number of sequences for a flank position (*n*/2)+1; maximum number of contiguous; non-conserved positions 50; minimum length of a block 5; and all gap positions allowed, where *n *is the total number of sequences in the aligned data set. These relaxed settings preserve large fractions of most alignments.

Bayesian phylogenetic inference was used to construct individual protein-family trees from the protein alignments and was carried out using MrBayes version 3.1.2 [29,30]. Ten alternative models of sequence change (Jones, Dayhoff, mtREV, MtMam, WAG, RtREV, CpREV, VT, Blosum, Equalin) were assigned a prior probability of 0.10 each. Inference was carried out using four Markov chains, run with default heating parameters and three of the four chains "heated". The heating parameter was fixed at 0.5. Protein sets with < 14 sequences were run for 1 million generations each, while sets with ≥ 14 sequences were run for 5 million generations each. All analyses used a burn-in of 50000 generations, and tree space was sampled every 100 generations.

### Reference *E. coli - Shigella *tree

The *E. coli - Shigella *reference tree was constructed using matrix representation with parsimony (MRP) [31]. The MRP input matrix was generated by recoding as character states all bipartitions among the 5282 individual Bayesian proteins trees that satisfy PP ≥ 0.95. The MRP reference tree was then computed from the matrix using the PARS program included in the PHYLIP package [48]. The reference tree was rooted on the edge connecting the B2 phylogenetic group and two group D strains with the remaining strains of the *E. coli - Shigella *species. This rooting is supported by a previous reconstruction of the *E. coli - Shigella *phylogenetic history which used closely related *Escherichia fergusonii *as an outgroup [32]. We accepted the resulting fully bifurcating topology as our reference *E. coli - Shigella *tree. Tree views were produced using Interactive Tree Of Life [49]

### Inference of discordant protein trees

To compare each of the 5282 protein trees with the reference topology we used the EEEP program [6,34] with a bootstrap collapse threshold of 95% (i.e. considering only nodes with PP ≥ 95%) and a strict reference tree ratchet (-rR). EEEP identifies discordance between a test tree and a reference tree and infers, when possible, edits paths which reconcile observed instances of discordance. Protein trees that were identified as topologically discordant with the *E. coli - Shigella *reference by EEEP were considered to provide *prima facie *evidence of the lateral transfer within the *E. coli - Shigella *clade of one or more genes belonging to the corresponding gene set. For protein trees which do not meet our criteria for discordance, we cannot reject the null hypothesis of concordance implying vertical inheritance.

### Inference of recombination breakpoints

For detection of recombination in nucleotide sequences, we implemented the two-phase strategy described by Chan *et al*. [33]. First, the amino acid alignments corresponding to the 5282 protein sets were computationally reverse-translated to nucleotide alignments using the corresponding nucleotide sequences from GenBank http://www.ncbi.nlm.nih.gov/. Three statistical measures [50-52] were then used to detect preliminary evidence of phylogenetic discrepancies within each set. Where a minimum of two of these three statistical tests revealed high significance of phylogenetic discrepancy across sites in an alignment (p-value < 0.1), potential recombination was inferred and that gene set was passed to the second phase, the phylogenetically based but computationally intensive DualBrothers [53]. We used the DualBrothers parameter value settings and the classification system described by Chan *et al*. [15] to identify sequence sets presenting clear evidence of recombination breakpoints within the gene boundaries. Inference of one or more recombination breakpoints has been interpreted as evidence of within-gene genetic transfer of one or more genes in the corresponding gene set [15].

### Network construction

A network representation of *E. coli *K12 transcriptional regulation was constructed using data obtained from RegulonDB (Release: 6.8, downloaded September 2010) [36]. More specifically, TF-target gene interactions were extracted from RegulonDB files *network_tf_gene.txt *and *network_tf_tf.txt*. All nodes in the resulting network represent genes; an edge is drawn from gene Gl to gene G2 if Gl encodes a TF that enacts regulation of G2. After removing RNA genes and obsolete genes, the final TRN consisted of 179 TF-encoding genes, 1533 target genes and 3, 804 regulatory interactions between them. A few of the TFs are active as heterodimers; in these cases, we included a node for each of the sub-unit encoding genes. Nine of the 179 TF-encoding genes present in the reconstructed *E. coli *K12 transcriptional regulation network did not go forward to phylogenetic analysis: six of these genes belong to protein sets of size *N *< 4, and thus do not contribute to meaningful phylogentic inference, while the remaining three were not annotated in the *E. coli *K12 MG1655 genome downloaded from NCBI.

### Definition of neighbour regulator

A custom python script was written to identify neighbour regulators, which we define as TF-encoding genes that regulate target genes which are encoded immediately adjacent on the chromosome. Applying this simple criterion, we identify 93 neighbour regulators. Adjacent heterodimer-encoding genes that regulate each other but no additional adjacent genes were excluded from the list of neighbour regulators.

### Functional analysis

For functional analysis of regulated target genes, all *E. coli *K12 protein-coding genes were assigned to a JCVI functional category. A role identifier (Mainrole) for each gene was retrieved from the JCVI Comprehensive Microbial Resource website http://cmr.jcvi.org/.

## List of abbreviations

(TF): Transcription Factor; (TRN): Transcriptional Regulatory Network; (LGT): Lateral Genetic Transfer; (PP): Posterior Probability; (SPR): Subtree Prune-and-Regraft; (ORB+): Observable Recombination Breakpoint positive; (ORB-): Observable Recombination Breakpoint negative.

## Authors' contributions

ES and MAR designed the research, analysed data and wrote the paper. ES performed the research. ES and MAR read and approved the final manuscript.
